# Divergent transcriptomic signatures in response to salinity exposure in two populations of an estuarine fish

**DOI:** 10.1111/eva.12799

**Published:** 2019-04-26

**Authors:** Ken M. Jeffries, Richard E. Connon, Christine E. Verhille, Theresa F. Dabruzzi, Monica T. Britton, Blythe P. Durbin‐Johnson, Nann A. Fangue

**Affiliations:** ^1^ Department of Biological Sciences University of Manitoba Winnipeg Manitoba Canada; ^2^ Anatomy, Physiology & Cell Biology, School of Veterinary Medicine University of California Davis California; ^3^ Wildlife, Fish & Conservation Biology University of California Davis California; ^4^ Bioinformatics Core Facility, Genome Center University of California Davis California; ^5^ Present address: Department of Ecology Montana State University Bozeman Montana; ^6^ Present address: Biology Department Saint Anselm College Manchester New Hampshire

**Keywords:** coastal fishes, extreme drought, genomic divergence, Sacramento splittail, single nucleotide polymorphism, transcriptome plasticity

## Abstract

In estuary and coastal systems, human demand for freshwater, climate change‐driven precipitation variability, and extreme weather impact salinity levels, reducing connectivity between mesohaline coastal fish populations and potentially contributing to genomic divergence. We examined gill transcriptome responses to salinity in wild‐caught juveniles from two populations of Sacramento splittail (*Pogonichthys macrolepidotus*), a species of conservation concern that is endemic to the San Francisco Estuary, USA, and the lower reaches of its tributaries. Recent extreme droughts have led to salinities above the tolerance limits for this species, creating a migration barrier between these populations, which potentially contributed to population divergence. We identified transcripts involved in a conserved response to salinity; however, the more salinity‐tolerant San Pablo population had greater transcriptome plasticity (3.6‐fold more transcripts responded than the Central Valley population) and a response consistent with gill remodeling after 168 hr of exposure to elevated salinity. The reorganization of the gill in response to changing osmotic gradients is a process critical for acclimation and would facilitate enhanced salinity tolerance. We detected an upregulation of receptors that control the Wnt (wingless‐type) cell signaling pathway that may be required for an adaptive response to increases in salinity, patterns not observed in the relatively salinity‐sensitive Central Valley population. We detected 62 single nucleotide polymorphisms (SNPs) in coding regions of 26 transcripts that differed between the populations. Eight transcripts that contained SNPs were associated with immune responses, highlighting the importance of diversity in immune gene sequences as a defining characteristic of genomic divergence between these populations. Our data demonstrate that these populations have divergent transcriptomic responses to salinity, which is consistent with observed physiological differences in salinity tolerance.

## 1 INTRODUCTION

Understanding how natural populations respond to changing environmental conditions is a fundamental research focus in ecology. Responses to environmental stressors observed in organisms are largely determined by a combination of phenotypic plasticity and adaptation (Crozier & Hutchings, 2014). Environmental stressors associated with climate change, such as changes in temperature, salinity, and incidences of disease, can alter the transcriptomes and phenotypes expressed in wild fish populations (Huang et al., 2016; Jeffries, Hinch, Sierocinski, Pavlidis, & Miller, 2014; Papakostas et al., 2012). However, plasticity in gene expression may differ among populations along environmental gradients (Dayan, Crawford, & Oleksiak, 2015). Furthermore, populations that are reproductively isolated can respond to different local selective pressures, which may facilitate adaptive divergence over time (Bradbury et al., 2010; Narum & Campbell, 2015; Papakostas et al., 2012). Genomic divergence can lead to differences in transcriptomic responses to environmental stressors between populations of wild fish.

Climate change and sea level rise will increase saltwater intrusion into freshwater systems, affecting organisms that rear in estuaries and lower reaches of coastal rivers (Chesney, Baltz, & Thomas, 2000). Furthermore, periods of extreme drought and low precipitation, which will be exacerbated by climate change (Karl & Trenberth, 2003), combined with increased water withdrawals for human use, will lead to decreased river outflows and increased salinities in estuaries, potentially impacting estuarine organisms (Knowles & Cayan, 2002; Martinho et al., 2007). Northern California is predicted to have decreased precipitation and river flows in the future due to climate change resulting in an increase in temperature and salinity in the San Francisco Estuary, USA (Cloern et al., 2011). Changes in salinity dynamics can effectively create migration barriers between coastal populations of mesohaline fishes resulting in reproductive isolation (Feyrer et al., 2015; Mahardja et al., 2015). Reproductive isolation may contribute to intraspecific variation in the response to salinity exposure suggesting adaptation to local environmental conditions (Brennan, Galvez, & Whitehead, 2015; DeFaveri & Merilä, 2014; Hasan et al., 2017; Papakostas et al., 2012; Scott, Rogers, Richards, Wood, & Schulte, 2004; Velotta, McCormick, & Schultz, 2015; Velotta et al., 2017; Verhille et al., 2016; Whitehead, Roach, Zhang, & Galvez, 2011). Characterization of fish responses to salinity can aid in predicting consequences of altered salinity levels on sensitive coastal and estuarine species (Komoroske et al., 2016).

**Figure 1:**
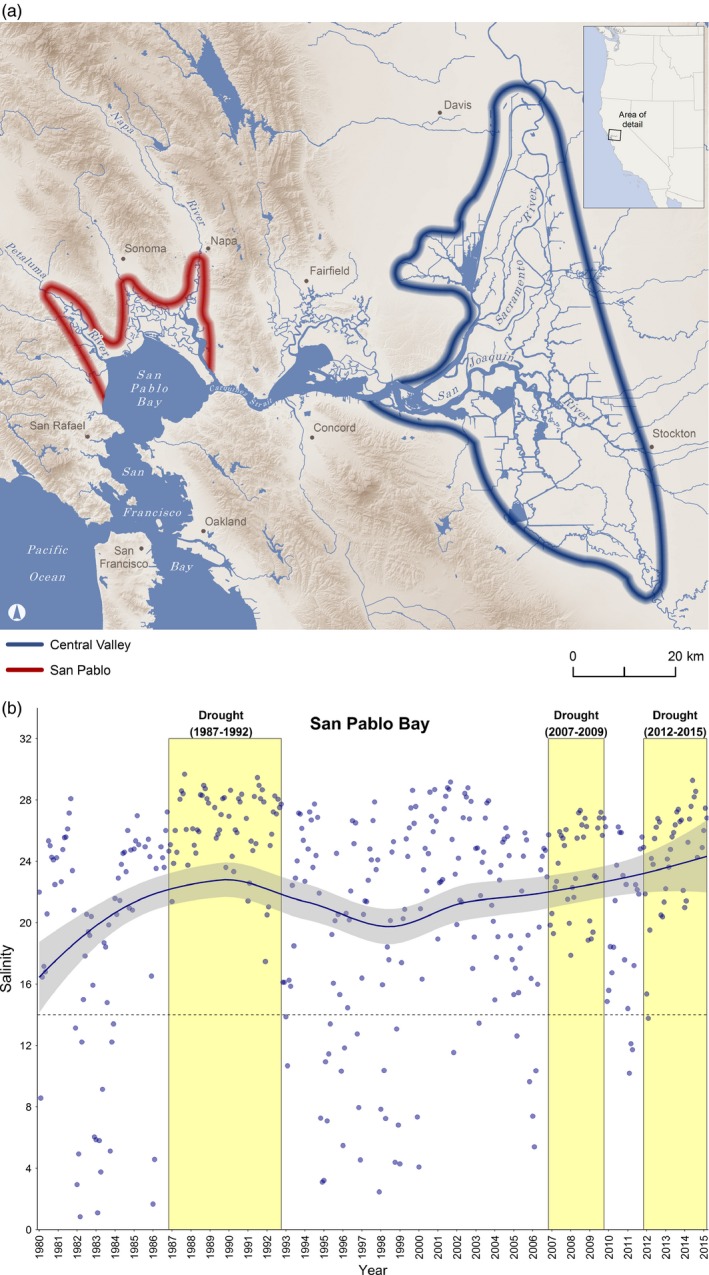
(a) Early rearing habitats for the San Pablo and Central Valley populations of Sacramento splittail (*Pogonichthys macrolepidotus*) in the San Francisco Estuary, California, USA (source data: CalAtlas. 2012. California Geospatial Clearinghouse. State of California. Available: http://atlas.ca.gov. Accessed: March 2012; Gesch et al. (2002); United States Geological Survey (USGS). 2017. National Hydrography Dataset. Accessed: March 2017. Available: https://nhd.usgs.gov/data.html). It should be noted that these distributions do not reflect the full extent of the semianadromous Sacramento splittail distribution in the San Francisco Estuary across lifestages as adult and subadult fish from both populations move into the estuary when conditions are appropriate and their distributions can overlap (Feyrer et al., 2015). (b) Monthly salinity measurements in San Pablo Bay near Carquinez Strait, the critical migration corridor separating spawning habitats of San Pablo and Central Valley populations, from 1980 to 2015. A reference line is included at 14 PSU as that was the salinity for the exposures in the present study and salinities greater than that lead to mortality in Sacramento splittail. Yellow‐shaded regions indicate periods of drought in California

We examined the effects of environmentally relevant salinities on wild‐caught juveniles (i.e., >1 year) from two populations of Sacramento splittail (*Pogonichthys macrolepidotus*), a semianadromous minnow endemic to the San Francisco Estuary, California, USA (Figure 1a). The Sacramento splittail had previously been listed as threatened under the United States Endangered Species Act; they are now currently considered a species of special concern in California. There are two genetically distinct populations of Sacramento splittail (Baerwald, Bien, Feyrer, & May, 2007; Baerwald, Feyrer, & May, 2008; Mahardja et al., 2015), the Central Valley population, which is larger and has a greater effective population size (i.e., up to 5 times larger; Mahardja et al., 2015), and the San Pablo population. Both populations rear as juveniles in their natal rivers and flood plains, followed by migration into the San Francisco Estuary as adults and subadults when conditions are appropriate, resulting in potential overlap of their distributions (Feyrer et al., 2015). As adults, the fish return to freshwater or low salinity water to spawn, and based on previous studies, during most years the two populations spawn in different locations leading to genetic differentiation (Baerwald et al., 2007; Feyrer et al., 2015; Mahardja et al., 2015). The San Pablo population experiences variable salinities due to saltwater intrusion into their natal rivers (ranging from 0 to 10 practical salinity units; PSU) during early rearing and are more salinity‐tolerant than the Central Valley population that rear in freshwater (Feyrer et al., 2015). Mortality occurs at lower salinities in the Central Valley population relative to the San Pablo population (Verhille et al., 2016), and neither population is found in the wild at salinities greater than 16 PSU (Feyrer et al., 2015). Low river outflows into the estuary during dry conditions contribute to salinities above the tolerance limits for this species and creates a migration barrier between these populations (Baerwald et al., 2007, 2008; Mahardja et al., 2015; Verhille et al., 2016). High salinities in the San Francisco Estuary may prevent gene flow between Sacramento splittail populations (Baerwald et al., 2007; Feyrer et al., 2015), and there has been evidence of genetic structure between these two populations for over a decade suggesting limited gene flow (estimated pairwise *R*_ST_ = 0.024–0.042 using microsatellites; Mahardja et al., 2015). During periods of drought, high salinities in the estuary are common (Figure 1b) suggesting that prolonged droughts may contribute to a lack of habitat connectivity and genomic divergence between the two populations.

We compared the gill transcriptome response to salinity in the two Sacramento splittail populations to test for molecular signatures consistent with differences in salinity tolerance. Characterization of transcriptome‐wide responses to changing environmental conditions provides critical information for managing coastal fishes of conservation concern (Connon, Jeffries, Komoroske, Todgham, & Fangue, 2018; Jeffries et al., 2016; Kalujnaia et al., 2007; Komoroske et al., 2016). We used RNA sequencing to examine the responses to short‐term and long‐term (i.e., 72 and 168 hr, respectively) exposures to elevated salinity (14 PSU) compared with a freshwater control group for each population. The exposure salinity and duration were chosen based on a previous laboratory holding study using these same Sacramento splittail populations that showed peak increases in plasma osmolality and gill Na^+^/K^+^‐ATPase activity after 72 hr of exposure to salinities of 14 PSU and divergent patterns in plasma osmolality, with the Central Valley individuals maintaining elevated osmolality after 168 hr (Verhille et al., 2016). Fourteen PSU were also shown to be the highest salinity that the Central Valley fish could experience without any mortality in the laboratory (Verhille et al., 2016). Our overall hypothesis was that there would be divergent transcriptomic responses to salinity exposure over a time course that reflect the differences in survival at elevated salinity between the two populations. We also hypothesized that the more salinity‐tolerant San Pablo population would show evidence of greater transcriptome plasticity that reflects variable salinity levels during early rearing conditions. We also compared transcript sequences to examine whether sequence differences occurred at frequencies high enough to suggest genomic divergence between the two populations. The goal of this study was to examine whether extreme drought‐related alterations to flow regimes and salinity dynamics in coastal systems may result in habitat fragmentation between populations of coastal fishes and contribute to genomic divergence.

## 2 MATERIALS AND METHODS

### 2.1 Fish collections

Gill samples for transcriptome sequencing were collected from a subset of individuals used in a previous study that examined the effects of salinity on physiological indices in Sacramento Splittail (detailed methods in Verhille et al., 2016). In brief, wild juvenile Sacramento splittail (>1 year) from the San Pablo population were collected from sites in the Napa River and juveniles from the Central Valley population were collected from multiple sites within their juvenile habitat range (Figure 1a) by the California Department of Fish and Wildlife. The salinity of the sites from where the San Pablo fish were collected ranged from 12.8 to 13.7 PSU, while the salinity of the sites where the Central Valley individuals were collected ranged from 0.1 to 5.5 PSU. Fish were transported to a holding facility at the University of California, Davis, and held in freshwater in 150‐L tanks. Fish were allowed to acclimatize to holding conditions for a minimum of 30 days before the salinity exposure experiments. Monthly salinity measurements are from locations near a constriction in the San Francisco Estuary (Carquinez Strait; data available at http://www.water.ca.gov; Figure 1b). This constriction is a critical migration corridor that enables habitat connectivity between the two populations of Sacramento splittail.

### 2.2 Salinity exposures

Fish were transferred to 20‐L tanks with flow‐through freshwater recirculation systems for the salinity treatments. There were two replicate tanks (*n* = 4–5 individuals per tank) for each population and treatment (i.e., total of 12 tanks). Fish were sampled in freshwater 24 hr after transfer, and these samples were used as the freshwater controls for each population as we were primarily interested in examining how the transcriptome changes over time due to exposure to salinity in these fish. Salinities were increased using Instant Ocean (Aquarium Systems) with a 6‐hr consistent ramp to the test salinity of 14 PSU and monitored using a YSI 556 MPS (YSI). Fourteen PSU were the highest salinity with no mortality in the Central Valley population during chronic exposures (Verhille et al., 2016) and are below the maximum salinity of 16 PSU where these fish are found in the wild (Feyrer et al., 2015). Fish were fed throughout the exposure period, except 24 hr prior to sampling. Fish remained at 14 PSU for 72 or 168 hr, after which they were euthanized in buffered tricaine methanesulfonate (MS‐222) and sampled for gill, muscle, and blood tissue. Blood samples were first centrifuged at 20,000 *g* for 5 min for plasma separation. Gill and blood plasma samples were immediately frozen in liquid nitrogen and then stored at −80°C. Additionally, the entire left gill arch was collected into a microcentrifuge tube filled with SEI buffer (250 mM sucrose, 10 mM Na2EDTA, 50 mM imidazole, pH 7.3) and frozen in liquid nitrogen for measuring gill Na^+^/K^+^‐ATPase activity.

Individuals from each population were genotyped (details in the supplementary data in Verhille et al., 2016) to determine whether the individuals were from the correct population (data not shown) prior to the transcriptomic assessments. Briefly, DNA was extracted from caudal fin tissue using a DNeasy 96 kit (QIAGEN Inc.) following manufacturer's protocols. Eighteen microsatellite markers were amplified for population genetic assignment: CypG3, CypG4, CypG23, CypG25, CypG35, CypG39, CypG40, CypG43, CypG45, CypG48, CypG52, CypG53, Pmac1, Pmac4, Pmac19, Pmac24, Pmac25, and Pmac35 (Baerwald & May, 2004; Mahardja, May, & Baerwald, 2012) following PCR and allele scoring procedures from Mahardja et al. (2015). The program STRUCTURE 2.3.3 (Pritchard, Stephens, & Donnelly, 2000) was used to assign individuals to their putative population and was performed for 10 iterations at *K* = 2 with all individuals and references included, no prior location information, a 500,000 burn‐in period, and 1 million Markov chain Monte Carlo repetitions under the assumption of admixture and correlated allele frequencies. Previously genetically assigned Sacramento splittail from Baerwald et al. (2007) and Mahardja et al. (2015) served as references for the present study. Replicate runs were averaged in CLUMPP 1.1.2 (Jakobsson & Rosenberg, 2007) with the FullSearch algorithm. An average *q*‐value of 0.8 was used as the threshold for distinguishing between individuals from the different populations and potential hybrids or unassigned individuals.

### 2.3 Transcriptome sequencing

Gill tissue samples were homogenized using a Qiagen TissueLyser in Buffer RLT Plus (RNeasy Plus Mini Kit), and 350 μl of the homogenate was used for RNA extraction on a QIAcube following manufacturer's protocols (Qiagen). There was a total of 32 fish used for the transcriptome sequencing (*n* = 16 from each population). The samples sizes from each time point for each population in the study were as follows: *n* = 4 for the freshwater control group; *n* = 6 for the 72 hr at 14 PSU group; and *n* = 6 for the 168 hr at 14 PSU group. The RNA quality was assessed using a Bioanalyzer (Agilent; RNA Integrity Numbers = 7.9–9.9 for all samples).

Sequencing was performed at the BGI@UC Davis facility in Sacramento, CA, USA. Total RNA was used to prepare cDNA libraries using KAPA Stranded mRNA‐Seq kits (KAPA Biosystems, Inc.) prior to sequencing. Each fish (i.e., 32 total) was individually barcoded with a unique adapter (NEXTflex DNA Barcodes; BIOO Scientific). Then, all 32 indexed libraries were pooled and sequenced on an Illumina HiSeq 2000 over six lanes. Mixed tissues from additional individuals (adults exposed to various stressors) were sequenced on another lane to improve transcriptome coverage for the de novo assembly. Sequencing was conducted to produce 100 base pair paired‐end reads generating an average of 34.8 (±5.9 *SD*) million raw pairs of reads per individual. Raw, demultiplexed reads were filtered and trimmed for low‐quality sequences using Sickle (https://github.com/ucdavis-bioinformatics/) to generate an average of 34.5 (±5.7 *SD*) million trimmed pairs (orphan single reads averaging 0.3 million per sample were discarded). Reads were normalized and assembled using Trinity v.2.0.6 (Haas et al., 2013) to produce a de novo transcriptome with 704,972 sequences (contigs). The raw assembly was filtered to remove ribosomal RNA (rRNA), low or unexpressed transcripts, and transcripts with no annotation (e.g., a blast hit, sequence description, or GO term) to a total of 118,853 transcript contigs, representing 28,534 genes.

Open reading frames (ORFs) were identified using TransDecoder v.2.0.1 (http://transdecoder.github.io), which included a blast search of zebrafish (*Danio rerio*) protein sequences (UniProt DANRE database) to maximize sensitivity for capturing ORFs that may have functional significance. Annotation was performed with Trinotate v.2.0.1, by blasting to subsets of the SWISS‐PROT and UniRef databases specific to Actinopterygii (ray‐finned) fishes. For contigs without blast hits to the subset databases, blasts were subsequently performed against the full Trinotate‐specific SWISS‐PROT and UniRef databases (https://data.broadinstitute.org/Trinity/Trinotate_v2.0_RESOURCES/). All steps in a standard Trinotate pipeline were run except RNAMMER as rRNA was already removed.

Reads were aligned to the reference transcriptome using BWA (Burrows‐Wheeler Aligner; Li & Durbin, 2010). A table of raw counts by transcript (from BWA alignments to the filtered assembly) was generated using Samtools v.1.2 idxstats (Li et al., 2009). Read counts for each contig were summed to gene level to generate a table of raw counts by gene for the differential expression analysis, which was conducted using edgeR (Robinson, McCarthy, & Smyth, 2010). A general linear model was run in edgeR with population and time as factors. A priori contrasts were designed to compare responses at 72 and 168 hr with the freshwater control group for each population. Genes were considered differentially expressed at a Benjamini–Hochberg‐corrected false discovery rate (FDR) <0.05. Due to large differences in the number and types of transcripts that responded between the two populations, we focused our interpretation of population‐specific responses to transcripts assigned to functional groups (i.e., Gene Ontology [GO] categories) using gene set enrichment analysis with EnrichR (Chen et al., 2013; accessed in September 2016). Only GO terms that had a minimum of four transcripts were used and were considered significantly enriched in the gene list at a Benjamini–Hochberg‐corrected FDR < 0.05. EnrichR only considers one copy of a gene for the analysis, and therefore, if there were more than one transcript annotated as the same gene in the gene list, the additional copies were ignored.

### 2.4 Sequence variation

Given the design of this comparative transcriptomics study, we opportunistically compared the transcript sequences from the individuals to identify mRNA transcripts with mutations that occur at different frequencies between the two populations. Single nucleotide polymorphisms (SNPs) were used to determine whether there was evidence of genomic divergence between the two populations of Sacramento splittail, consistent with previous studies that used microsatellites (Baerwald et al., 2007, 2008; Mahardja et al., 2015). Our SNP analysis focused on mutations within the coding region of the transcripts as we were interested in knowing whether there are SNPs within the transcripts that may contribute to functional differences in the responses to salinity. The longest transcript within each gene contig was selected for identifying SNPs. Reads were aligned to the assembled reference transcriptome (i.e., the 28,534 sequence FASTA file) with BWA, and Picard MarkDuplicates was run (Picard tools v.1.139, https://broadinstitute.github.io/picard/). The SNPs were called with FreeBayes v.0.9.18‐1 (Garrison & Marth, 2012), and the effects of SNPs were determined with SnpEff v.4.1l (Cingolani et al., 2012). We only considered SNPs in transcripts that were present in at least 75% of the individuals and had a minimum read depth of 500. We used pcadapt (Luu, Bazin, & Blum, 2017) to look for evidence of population structure and to test for outlier SNPs after filtering out SNPs with a minor allele frequency <0.05. After running an initial PCA, we used the “score plot” approach to determine the number of PCs to use for the outlier analysis. The best evidence of population structure was seen in the first 2 PCs; therefore, the subsequent outlier analysis was limited to 2 PCs. Outliers were considered significant using the Mahalanobis distance at a *q*‐value <0.05. We also used BayeScan to test for outlier SNPs (Foll & Gaggiotti, 2008). Using BayeScan, SNPs were considered significant at a *q*‐value <0.05. When SNPs were significant using both approaches (i.e., BayeScan and pcadapt), we considered them to be under selection and contribute to genomic differentiation between the two populations. We tested for genetic differentiation by estimating the pairwise *F*_ST_ using the Weir and Cockerham (1984) method for calculating *F*_ST_ in the program HIERFSTAT (Goudet, 2005).

### 2.5 Physiological parameters

Plasma osmolality was measured on plasma samples that were equilibrated to room temperature using a Vapro™ Vapor Pressure Osmometer (Model 5600) equipped with a mini sample holder to allow for small (2 µl) sample volumes. Dorsal skeletal muscle samples weighing ~100 mg were collected for muscle water content analysis. Wet mass of muscle samples was measured on a 4‐digit analytical balance. Samples were then desiccated for 7 days at 55°C and then reweighed. Muscle moisture was calculated as the percent of wet mass lost with desiccation.

The Na^+^/K^+^‐ATPase activity was quantified by measuring the rate of NADH loss through enzymatically coupling Na^+^/K^+^‐ATPase catalyzed ATP hydrolysis with the oxidation of NADH to NAD catalyzed by lactate dehydrogenase (McCormick, 1993). Gill samples were thawed on ice; then, the SEI buffer was pipetted out and replaced with 0.5–1 ml of fresh chilled 0.5% SEID buffer (250 mM sucrose, 10 mM Na2EDTA, 50 mM imidazole, 0.5% Na Deoxycholic acid, pH 7.3) depending on the size of the sample. Each sample was homogenized using a Polytron Homogenizer (KINEMATICA AG, Lucerne, Switzerland) for 30 s then centrifuged for 30 s at 4°C and 5,000 *g*. The supernatant was pipetted off and stored on ice prior to enzyme activity readings. Ten‐minute spectrophotometric kinetic reads of 340 nm absorbance (25°C, Synergy HT microplate reader, BioTek) were performed on triplicate 10 µl volumes of samples loaded onto microplates then combined with 200 µl of ouabain+ or ouabain− assay solutions warmed to 25°C. Total protein was quantified using a commercial test kit (BCA protein assay kit; Thermo Scientific) according to the bicinchoninic acid technique (Smith et al., 1985). The Na^+^/K^+^‐ATPase enzyme activity was standardized to total protein among samples. Na^+^/K^+^‐ATPase activities (µmol ADP mg protein^−1^ hr^−1^) were calculated as the ouabain inhibited fraction of total ATP hydrolysis and conversion of NADH to NAD^+^. Differences in the physiological indices were analyzed with a two‐factor ANOVA with time and population as factors. Tukey's HSD post hoc tests were performed, and values were considered significant at *p* < 0.05 after correcting for multiple testing.

## 3 RESULTS

### 3.1 Transcriptome sequencing

**Figure 2:**
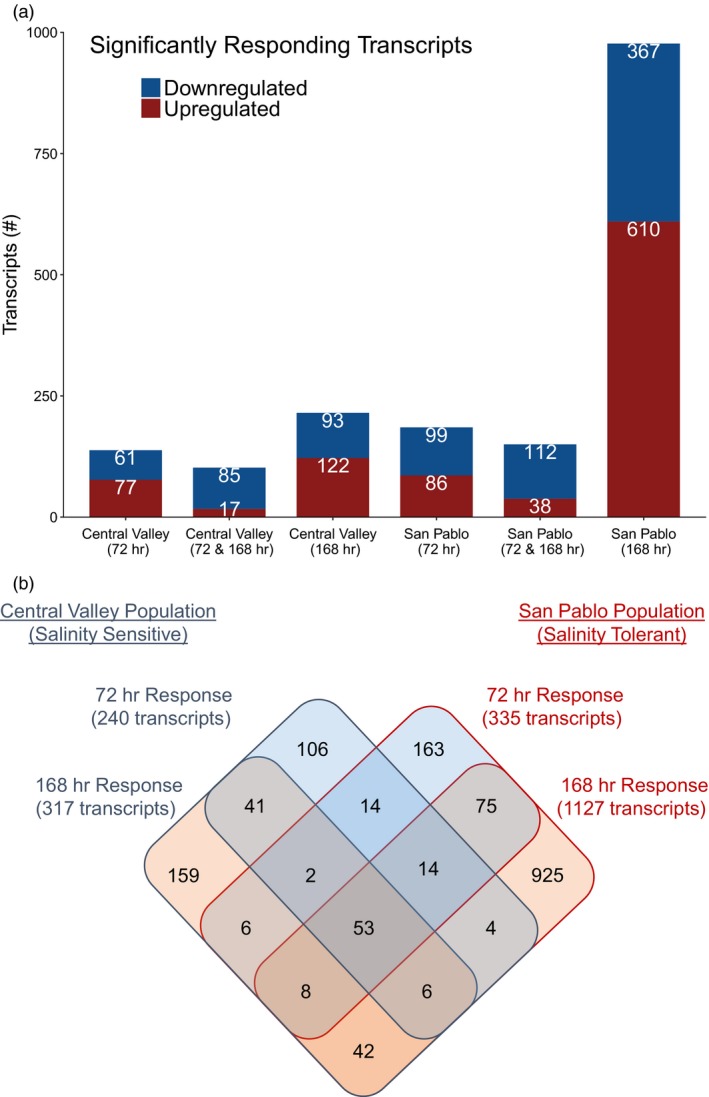
(a) Number of upregulated and downregulated transcripts (false discovery rate <0.05) relative to the freshwater control group in two populations (the salinity‐sensitive Central Valley and the salinity‐tolerant San Pablo) of Sacramento splittail (*Pogonichthys macrolepidotus*) at 72 and 168 hr of exposure to elevated salinity relative to the freshwater control group; (b) venn diagram of the number of transcripts that were differentially expressed in common between the different populations at the different time points

There were 53 transcripts differentially expressed that were common in both the short‐term (72‐hr) response and the longer‐term (168‐hr) acclimation response relative to the freshwater control groups at FDR < 0.05 (Figure 2). Nine of these common transcripts were upregulated, including two Cytochrome P450 proteins (1A1 and 1A3), two transcripts involved in protein transport, and two transcripts annotated as inositol‐3‐phosphate synthase 1‐A, which plays an important role in osmoregulation. The remaining 44 common transcripts were downregulated, and many were involved in ion transport and homeostasis, including seven transcripts annotated as sodium/potassium‐transporting ATPase subunit alpha‐1. Of the other common downregulated transcripts, two were annotated as band 3 anion exchange protein, four as chloride channel protein 2, one as ornithine decarboxylase, one as solute carrier 12 member 3, and one as prolactin receptor. Complete lists of differentially expressed transcripts at FDR < 0.05 for both populations are provided in the Supplementary materials (Table S1).

**Figure 3:**
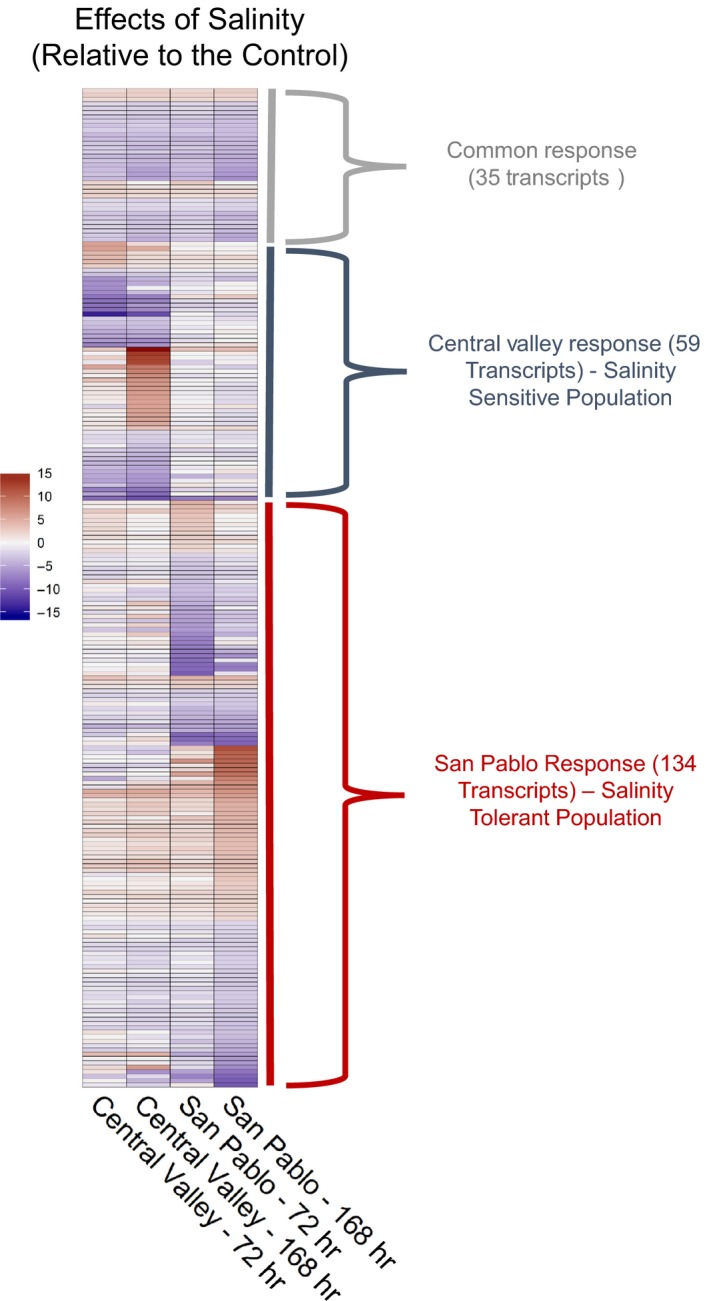
Heatmap of differentially expressed transcripts (false discovery rate [FDR] <0.01) in two populations (the salinity‐sensitive Central Valley and the salinity‐tolerant San Pablo) of Sacramento splittail (*Pogonichthys macrolepidotus*) at 72 and 168 hr of exposure to elevated salinity. Transcripts are presented as log2 fold change that were significant at FDR < 0.01 and a minimum 2‐fold change (number of transcripts that were differentially expressed are in parentheses) in expression relative to the freshwater control group. Only a subset of the transcripts was used for the heatmap to improve visualization of the patterns, the number of transcripts that were significant at FDR < 0.05 is available in Figure 2

There were population‐specific responses associated with the 72‐hr and 168‐hr salinity exposures (Figure 3). At both time points, the San Pablo fish had more transcripts respond to the salinity treatments compared with the Central Valley fish, suggesting enhanced transcriptome plasticity in the more salinity‐tolerant San Pablo population (Figure 2b). This was more evident at 168 hr, where there were 977 significant transcripts in the San Pablo population in contrast to 215 transcripts in the Central Valley population. Within each population‐specific response, 150 and 102 transcripts for the San Pablo and Central Valley populations, respectively, showed common response patterns at 72 and 168 hr of exposure relative to the freshwater control.

**Figure 4:**
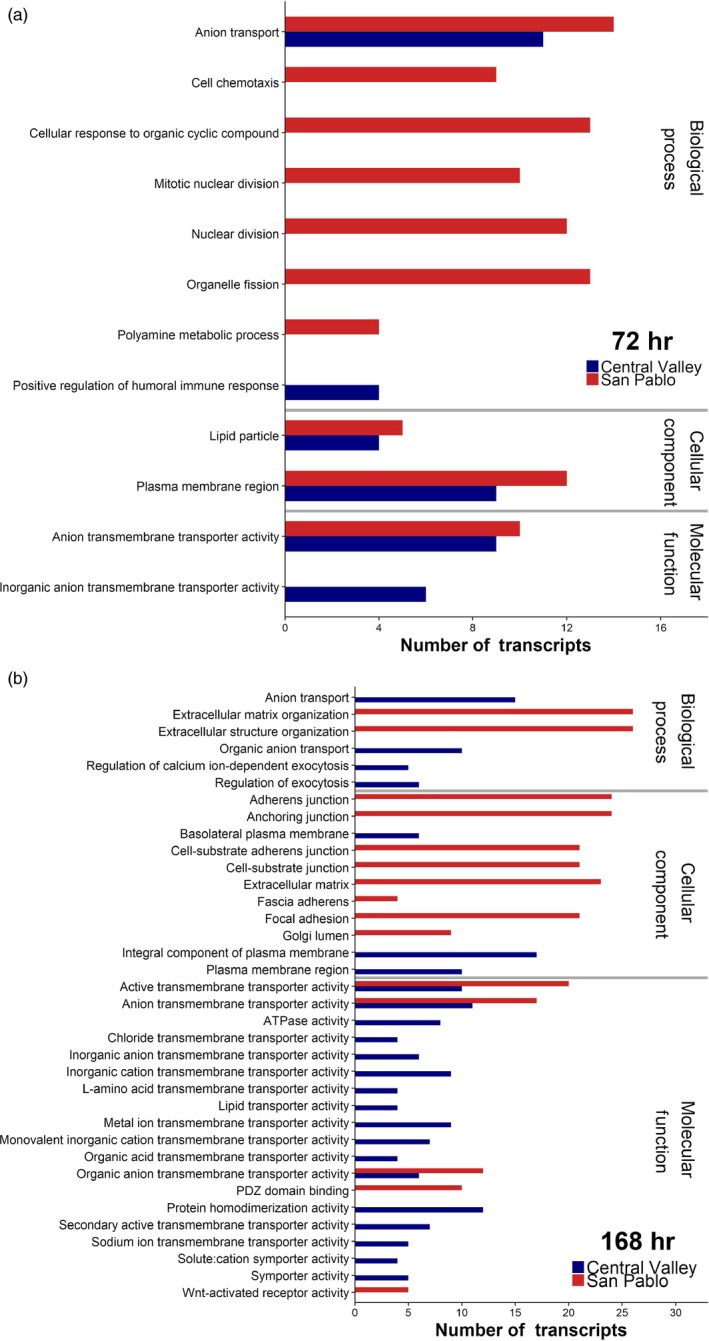
Gene ontology (GO) terms enriched in the significant gene lists from two populations of Sacramento splittail (*Pogonichthys macrolepidotus*) at (a) 72 hr and (b) 168 hr of exposure to elevated salinity. Only GO terms from the gene set enrichment analysis significant at a FDR < 0.05 that had more than four transcripts were considered

**Figure 5:**
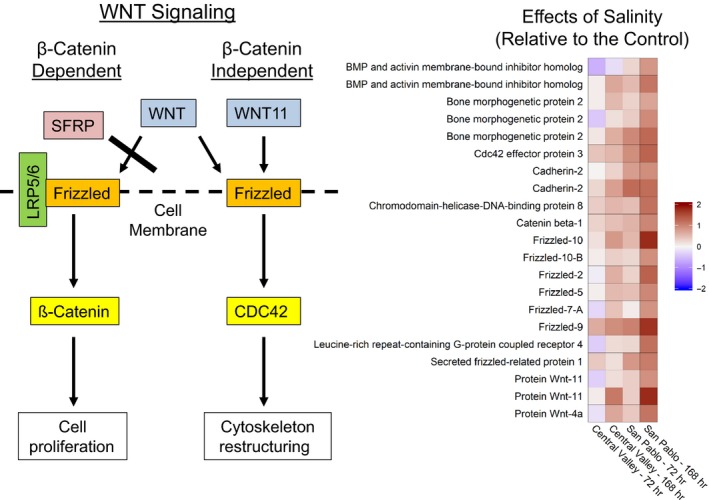
Heatmap of upregulated expressed transcripts (false discovery rate < 0.05) involved in the Wnt (Wingless‐type) signaling pathway in the San Pablo population of Sacramento splittail (*Pogonichthys macrolepidotus*) at 168 hr of exposure to elevated salinity relative to the freshwater control group. Expression of genes is presented as log2 fold change for transcripts. Protein Wnt‐11 is a main ligand, and CDC42 (CDC42 effector protein 3) is a key regulator of the planar cell polarity branch of the β‐catenin‐independent Wnt signaling pathway that is associated with cytoskeleton remodeling (SFRP, Secreted frizzled‐related protein; Frizzled, Frizzled receptor; LRP5/6, low‐density lipoprotein receptor‐related protein 5 and 6 complex)

After 72 hr, both populations had altered expression of transcripts associated with ion and general transport across the cell membrane (Figure 4a), a required response in gills to contend with osmotic gradients. The response patterns between the two populations diverged during the 168 hr of salinity exposure (Figure 4b). Many of the 977 transcripts that were significant in the San Pablo fish may be associated with processes involved in acclimation. For example, the San Pablo population had many significantly responding transcripts associated with cellular turnover and proliferation (e.g., GO categories Mitotic nuclear division, Organelle fission), potentially consistent with cellular remodeling. Additional details of the Gene Set Enrichment Analysis results are available in Table S2. The San Pablo population had many transcripts respond at 168 hr that are involved in the extracellular matrix organization (e.g., upregulation of Matrix metalloproteinase‐11, Matrix metalloproteinase‐16, and Matrix metalloproteinase‐21), cell adhesion, and cell junctions (e.g., GO categories Extracellular matrix, Adherens junction, Focal adhesion, Wnt‐activated receptor activity). Interestingly, there was an upregulation of Metalloproteinase inhibitor 3 in the San Pablo fish, which is involved in repressing the expression of some matrix metalloproteinases including Matrix metalloproteinase‐13. The upregulation of Metalloproteinase inhibitor 3 coincided with a downregulation of Matrix metalloproteinase‐13. Both populations had differential expression of several transcripts involved in Wnt (wingless‐type) cell signaling. However, the San Pablo population at 168 hr responded by upregulating key transcripts (Secreted frizzled‐related protein 1, Protein Wnt‐11, Protein Wnt‐4A, Catenin beta‐1; CDC42 effector protein 3) and associated receptors (Frizzled 2, 5, 7‐A, 9, 10, and 10‐B) involved in Wnt signaling (Figure 5). Lastly, the San Pablo population had a downregulation of Suppressor of cytokine signaling 2 at 168 hr.

### 3.2 Sequence variation

**Figure 6:**
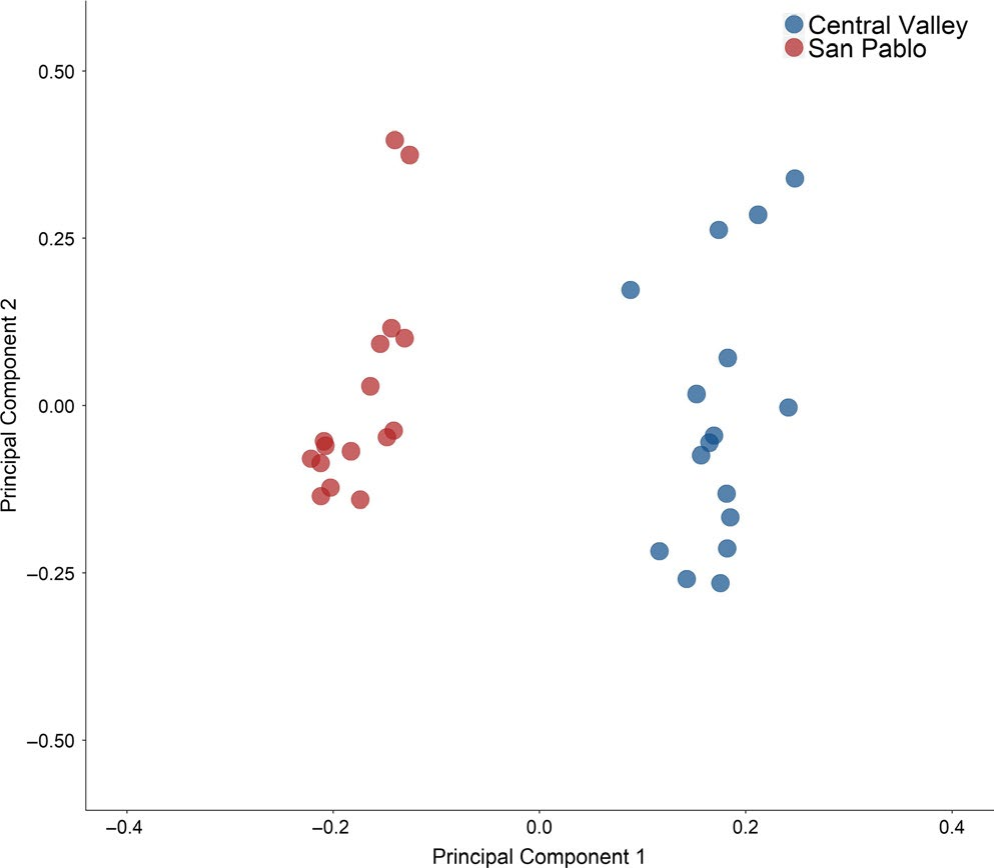
Position of each fish (*n* = 32) from two populations (San Pablo or Central Valley) of Sacramento splittail (*Pogonichthys macrolepidotus*) along the first two principal component (PC) axes from the principal component analysis conducted on the genotype assigned from 82,461 SNPs detected in the coding regions of the transcripts

We detected 181,059 SNPs that occurred in the transcripts from the two populations. Using the full set of 181,059 SNPs, we calculated a pairwise F_ST_ of 0.064. After filtering out the SNPs with a minor allele frequency <0.05, we were left with 82,461 SNPs within the coding region of the transcripts to test for outliers. The PCA conducted on the filtered set of SNPs clearly separated the two populations along the PC1 axis, which explained 7.06% of the variation in the data (Figure 6). Another 4.72% of the variation in the data was explained by PC2, the relatively large amount of variation explained by PC2 was largely driven by the 5 fish on the most positive end of the PC2 axis (two and three fish from the San Pablo and Central Valley populations, respectively). The SNPs that were significant along both PC1 and PC2 with all 32 fish and with those 5 fish removed are available in the Supplementary Materials (Table S3). Because the PC1 axis showed the most separation between the populations and our interests were in SNPs that may contribute to differentiation between the two populations, we limit our interpretations to SNPs that were significant along PC1. Using pcadapt, we detected 147 SNPs that were significantly associated with PC1 at a FDR < 0.05 (Table S3). We calculated a pairwise *F*_ST_ of 0.250 using these 147 SNPs. Using BayeScan, we detected 74 SNPs that were significantly different between the two populations at *q* < 0.05. There were 62 SNPs that were significant and were common using both pcadapt and BayeScan approaches. Of these, 37 were nonsynonymous SNPs, 23 were synonymous SNPs, and 2 were stop gained SNPs (Table S3). There were 62 potential outlier SNPs that were identified; however, these occurred in 26 unique transcripts. Eight of the unique transcripts containing outlier SNPs were associated with immune responses. In particular, SNPs were found in transcripts involved in the regulation of the adaptive immune response (e.g., Major histocompatibility complex class I‐related gene protein; Class I histocompatibility antigen, F10 alpha chain; 2 and 4 unique transcripts, respectively, were annotated as these genes), T‐cell receptor alpha chain V region RL‐5 and Protein NLRC3. There were 7 SNPs in one transcript annotated as Interferon‐induced very large GTPase 1. Lastly, there were SNPs in transcripts that may have a role in ion transport (solute carrier family 22 member 6, Serine/threonine‐protein kinase WNK3), an oxidative stress response (Thioredoxin), and cytoskeleton restructuring (Protein kinase C and casein kinase substrate in neurons protein 1). Of these, only Protein kinase C and casein kinase substrate in neurons protein 1 was a nonsynonymous SNP.

### 3.3 Physiological indices

**Figure 7:**
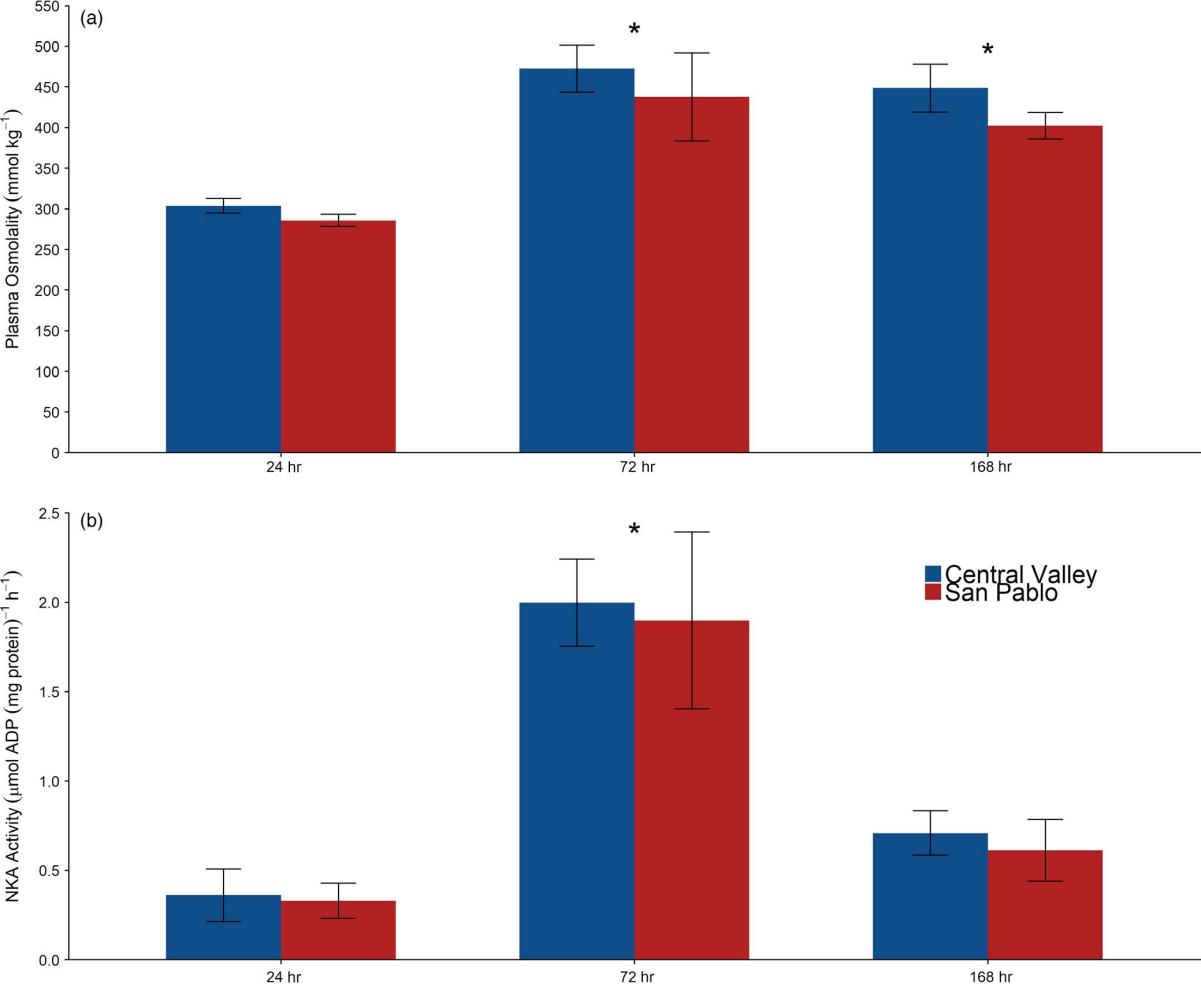
Mean (±*SE*) values for (a) plasma osmolality and (b) gill Na^+^/K^+^‐ATPase (NKA) activity for two populations of Sacramento splittail (*Pogonichthys macrolepidotus*) after 72 and 168 hr of exposure to saltwater. Statistical differences from the 24‐hr freshwater control group are marked with an asterisk

There was an increase in Na^+^/K^+^‐ATPase enzyme activity at 72 hr and plasma osmolality at 72 and 168 hr in the salinity treatments relative to the freshwater control group (Figure 7). The Na^+^/K^+^‐ATPase enzyme activity at 72 hr was also significantly different than the 168‐hr group. There were no differences in muscle moisture content. Despite large differences in the transcriptome‐wide responses to the salinity treatment, we did not detect population‐specific responses in the physiological indices. Although the Central Valley population tended to have higher plasma osmolality, this was not statistically significant.

## 4 DISCUSSION

Transcriptome sequencing is a valuable approach to study mechanisms underlying essential conservation biology issues, such as stress tolerance, local adaptation, and population divergence (Connon et al., 2018; Gibbons, Metzger, Healy, & Schulte, 2017; Jeffries et al., 2016; Komoroske et al., 2016; Velotta et al., 2017; Zhang et al., 2015). The present study uses this approach to assess population‐specific responses to elevated salinity in a nonmodel fish species. We were able to show (a) evidence of population‐specific transcriptomic responses to a common salinity stressor; (b) that the enhanced salinity tolerance in the San Pablo population is associated with a transcriptome signature consistent with gill remodeling required for salinity acclimation; and (c) that there was sequence variation in transcripts associated with immune responses, which supports previous studies that demonstrate variation in genes involved in immune responses are major signatures of genomic divergence between fish populations (Dionne, Miller, Dodson, Caron, & Bernatchez, 2007; Eizaguirre, Lenz, Kalbe, & Milinski, 2012; Evans, Neff, & Heath, 2010; Miller, Kaukinen, Beacham, & Withler, 2001; Zhang et al., 2015; Zueva et al., 2014).

We identified a suite of transcripts involved in a conserved response to salinity in the two populations. There was a downregulation of some Sodium/potassium‐transporting ATPase transcripts, which has been shown in other species after exposure to elevated salinities (Brennan et al., 2015; Komoroske et al., 2016; Velotta et al., 2017) and may suggest either a downregulation of freshwater isoforms or sufficient protein level expression resulting in regulatory feedback on transcription. Previous work on other fishes has shown higher expression of chloride channel protein 2, prolactin receptor, solute carrier 12 member 3, Suppressor of cytokine signaling 2, and ornithine decarboxylase in freshwater (Barrio et al., 2016; Evans & Somero, 2008; Komoroske et al., 2016; Lai et al., 2015; Lam et al., 2014; Whitehead et al., 2011), which is consistent with the observed downregulation at higher salinity in the present study. Furthermore, Suppressor of cytokine signaling 2 has been suggested to be associated with salinity adaptation in marine fishes (Barrio et al., 2016; Dalongeville, Benestan, Mouillot, Lobreaux, & Manel, 2018). Fish in the present study were held under common conditions to control for other environmental factors that can influence transcriptome patterns; therefore, the divergent responses to salinity are likely partially associated with genomic differences between the two populations. However, the role of salinity exposure histories prior to collection of fish from the wild and potential transgenerational effects may have also influenced the transcriptome patterns. Additionally, given that most of the outlier SNPs detected in this study were not in transcripts associated with a response to an increase in salinity, plasticity may have played the largest role in the differences in the transcriptome responses in the present study. Future research should attempt to control for transgenerational effects in order to truly assess the influence of genotype on the phenotypes expressed in these populations.

There was a suite of transcripts involved in distinct population‐specific transcriptome responses to salinity. The San Pablo population, which rears in rivers that can have significant interannual variation in saltwater intrusion (Baerwald et al., 2007; Mahardja et al., 2015), had more transcripts respond to salinity exposure (1.4 and 3.6 times more during the short‐term and acclimation response, respectively) than the Central Valley population, consistent with increased transcriptome plasticity. Greater transcriptome plasticity in a population may reflect increased environmental heterogeneity (Meier et al., 2014) or higher salinities (Hasan et al., 2017) in their natal habitats. The San Pablo population, which experiences variable and generally higher salinities than the Central Valley population due to saltwater intrusion in the rivers during early rearing (Feyrer et al., 2015), may require enhanced transcriptome plasticity to respond to the changes in salinity. Furthermore, the greater number of transcripts that responded in the San Pablo population at 168 hr of salinity exposure may represent a critical acclimation response that leads to enhanced salinity tolerance relative to the Central Valley population.

Interestingly, we detected no statistical differences in plasma osmolality, muscle moisture, or gill Na^+^/K^+^‐ATPase activity between the two populations. This may have been due to a lack of statistical power because of low sample sizes (*n* = 4–6 per treatment) as differences have been reported previously from the same study when larger sample sizes were assessed (Verhille et al., 2016). Despite no differences in the responses of these endpoints for the fish used in the present study, there are differences in the upper tolerance limits for salinity for the two populations (Verhille et al., 2016) and large differences in the transcriptome‐wide response. This may suggest that different molecular phenotypes can achieve the same functional response for these osmoregulatory indices in the two populations of Sacramento splittail. However, the transcriptome‐wide response patterns observed in the present study are consistent with the differences in salinity tolerance in the two populations of Sacramento splittail.

Gill remodeling can be an adaptive response to various environmental stressors that may be fairly widespread among fishes (Nilsson, 2007). Remodeling of the gills in response to salinity acclimation has been shown to contribute to increased number of mitochondrial‐rich cells, alteration of the apical surface of gill cells, and increased Na^+^/K^+^‐ATPase activity, which are all necessary to facilitate ion excretion in saltwater (Foskett, Logsdon, Turner, Machen, & Bern, 1981; Hirose, Kaneko, Naito, & Takei, 2003; Hwang & Lee, 2007; Karnaky, Ernst, & Philpott, 1976; Sinha, Matey, Giblen, Blust, & Boeck, 2014). Altered expression of genes associated with cytoskeleton reorganization is involved in transcriptome responses to salinity changes in several other fishes (Evans & Somero, 2008; Gibbons et al., 2017; Lai et al., 2015; Lam et al., 2014) and has been linked to gill cellular remodeling (Whitehead, Zhang, Roach, & Galvez, 2013). Additionally, the gill cellular remodeling occurs faster in euryhaline fishes when compared to more stenohaline species (Whitehead et al., 2013). Increased levels of transcripts associated with extracellular matrix and cell adhesion in the fish from the San Pablo population are consistent with gill restructuring during acclimation. Gill restructuring in response to higher salinity levels suggests adaptive plasticity that would facilitate enhanced salinity tolerance in the San Pablo population.

The San Pablo population showed activation of the Wnt signaling pathway during acclimation. This included increased expression of transcripts in the frizzled family that function as receptors for secreted Wnt proteins and play a critical role in development and cell proliferation (Huang & Klein, 2004). Changes in the expression of receptors that control regulatory networks are key contributors to population divergence in fishes (Di Poi, Bélanger, Amyot, Rogers, & Aubin‐Horth, 2016). Our data support this assertion as the salinity‐tolerant San Pablo population had upregulated receptors that control the Wnt signaling pathway. Upregulation of the Wnt signaling pathway may be required for an adaptive response to increases in salinity and did not occur in the relatively salinity‐sensitive Central Valley population. The Wnt signaling pathway is critical to adult tissue maintenance, remodeling and regeneration, embryo development, and many cellular processes that include cell motility and cytoskeleton restructuring (Angers & Moon, 2009; Lai, Chien, & Moon, 2009), and is associated with cellular remodeling in fishes (Cui et al., 2014). Protein Wnt‐11 is a main ligand, and CDC42 effector protein 3 is a key regulator, of the planar cell polarity branch of the β‐catenin‐independent Wnt signaling pathway that is associated with cytoskeleton remodeling (Angers & Moon, 2009; Lai et al., 2009). Both Protein Wnt‐11 and CDC42 effector protein 3 were upregulated in the San Pablo population fish.

Fish populations show the capacity to adapt to local environmental conditions throughout their natural ranges (Eliason et al., 2011; Hecht, Matala, Hess, & Narum, 2015; Taylor, 1991). Local salinities may drive adaptive divergence (Brennan et al., 2018; Dennenmoser, Vamosi, Nolte, & Rogers, 2017); however, significant variation often occurs in genomic regions associated with immune responses (Dionne et al., 2007; Eizaguirre et al., 2012; Evans et al., 2010; Miller et al., 2001; this study). Between populations, selection on genomic regions associated with immune function is a significant driver of adaptive divergence (Zueva et al., 2014). The genomic diversity in immune response genes may be driven by local pathogen communities in addition to differences in life‐history characteristics (e.g., migratory strategies) between populations (Miller et al., 2001). The SNPs detected in Sacramento splittail that were significant in our analyses largely occurred in transcripts associated with an immune response, in particular an adaptive immune response. The role of major histocompatibility complex (MHC) diversity among populations is well established in fishes (Dionne et al., 2007; Eizaguirre et al., 2012; Evans et al., 2010; Miller et al., 2001), and our data support this trend as we detected variation in MHC‐related genes between the two Sacramento splittail populations. Our data suggest that there is a signal of genomic divergence between the two populations of Sacramento splittail; however, the strongest patterns were related to immune function rather than a salinity response.

Sacramento splittail evolution and population structure will likely be heavily determined by future climate conditions (Feyrer et al., 2015). Climate variability is predicted to increase due to climate change (Karl & Trenberth, 2003), which may lead to frequent severe droughts in coastal regions. During dry years and periods of drought, such as those that have occurred recently in California, salinity levels within the estuary increase to those above the tolerance limits for Sacramento splittail, therefore reducing connectivity between the populations. High salinities in the San Francisco Estuary may prevent gene flow between Sacramento splittail populations (Baerwald et al., 2007; Feyrer et al., 2015), and this lack of habitat connectivity during periods of high salinity may have contributed to the divergent transcriptomic responses observed in the present study. The Central Valley population is likely more susceptible to future saltwater intrusion based on upper salinity tolerances (Verhille et al., 2016) and reduced transcriptome plasticity compared with the San Pablo population. Conversely, the San Pablo population is potentially better adapted for future climate change scenarios that may lead to greater saltwater intrusion into coastal rivers, but has a smaller effective population size and is therefore more susceptible to genetic drift and extirpation. Our data demonstrate that these populations of Sacramento splittail have divergent transcriptomic responses to salinity, suggestive of local adaptation; however, persistence of these populations and this species will depend on sufficient minimal freshwater flows into the estuary that keep salinity levels below tolerance thresholds in critical habitats. Precipitation variability and extreme weather conditions in the future, in addition to greater human demand for freshwater, will contribute to reduced river flows leading to saltwater intrusion into coastal rivers and increased salinity variability in estuaries, potentially reducing habitat connectivity between mesohaline coastal fish populations and contributing to population divergence over time.

## CONFLICT OF INTEREST

None declared.

## AUTHOR CONTRIBUTION

K.M.J., C.E.V., R.E.C., and N.A.F. designed the study. C.E.V. and T.F.D. conducted the salinity exposure experiments. K.M.J processed the samples and designed the sequencing study. M.T.B., B.P.D.‐J., and K.M.J. analyzed the data. K.M.J. wrote the first draft of the manuscript, and all authors contributed to the final version.

## Data Availability

Raw sequence data are available through the National Center for Biotechnology Information Sequence Read Archive (accession #SRP077066).
